# Establishment and application of TSDPSO-SVM model combined with multi-dimensional feature fusion method in the identification of fracture-related infection

**DOI:** 10.1038/s41598-023-46526-w

**Published:** 2023-11-10

**Authors:** Xiaofeng Hu, Jianmin Chen, Xiaofei Zheng, Jianmei Li, Mingwei Zhou

**Affiliations:** Department of Orthopaedics, Jinling Hospital, School of Medicine, Nanjing University, No. 34, Lot 34, Changfu Street, Qinhuai District, Nanjing, Jiangsu Province China

**Keywords:** Infectious diseases, Trauma

## Abstract

Fracture-related infection (FRI) is one of the most common and intractable complications in orthopedic trauma surgery. This complication can impose severe psychological burdens and socio-economic impacts on patients. Although the definition of FRI has been proposed recently by an expert group, the diagnostic criteria for FRI are not yet standardized. A total of 4761 FRI patients and 4761 fracture patients (Non-FRI) were included in the study. The feature set of patients included imaging characteristics, demographic information, clinical symptoms, microbiological findings, and serum inflammatory markers, which were reduced by the Principal Component Analysis. To optimize the Support Vector Machine (SVM) model, the Traction Switching Delay Particle Swarm Optimization (TSDPSO) algorithm, a recognition method was proposed. Moreover, five machine learning models, including TSDPSO-SVM, were employed to distinguish FRI from Non-FRI. The Area under the Curve of TSDPSO-SVM was 0.91, at least 5% higher than that of other models. Compared with the Random Forest, Backpropagation Neural Network (BP), SVM and eXtreme Gradient Boosting (XGBoost), TSDPSO-SVM demonstrated remarkable accuracy in the test set ($\chi^{ 2} = 29.17, \,50.46, \,56.66,\,35.88, P < 0.01$). The recall of TSDPSO-SVM was 98.32%, indicating a significant improvement ($\chi^{2} = 91.78,\, 107.42, \,135.69, P < 0.01$). Compared with BP and SVM, TSDPSO-SVM exhibited significantly superior specificity, false positive rate and precision ($\chi^{2} > 3.84, P < 0.05)$. The five models yielded consistent results in the training and testing of FRI patients across different age groups. TSDPSO-SVM is validated to have the maximum overall prediction ability and can effectively distinguish between FRI and Non-FRI. For the early diagnosis of FRI, TSDPSO-SVM may provide a reference basis for clinicians, especially those with insufficient experience. These results also lay a foundation for the intelligent diagnosis of FRI. Furthermore, these findings exhibit the application potential of this model in the diagnosis and classification of other diseases.

## Introduction

With the rapid advancement in industry and transportation in recent years, such accidental traumas as car accidents and falls from heights, as well as trauma caused by machinery, have been increasing with each passing year. According to statistics, there are as many as 5 million patients with fractures in the United States every year. This number is set to surge to 60 million worldwide. In patients with open fractures, the presence of a wound indicates a high risk of fracture contact and soft tissue injury, which may induce post-fracture infection^1^. Fracture-related infection (FRI) is a common, costly, destructive, and difficult-to-treat complication in orthopedics. The research group of the 2018 International Consensus Conference reported that the incidence of infection in all subspecialties of orthopedics in North America ranged from 0 to 50.0%^2^. Postoperative infections may still be present after surgical treatment of open fractures. According to research results, the internal fixation infection rate was 0–2% in non-open fractures and Type I open fractures, and that was 2–7% and 10–25% in Type II open fractures and Type III open fractures, respectively^3^. Even if it is not an open fracture, there is a possibility of blood-borne infection. It has even been reported that after the reclassification of type III open fractures based on Gustilo classification, the infection rate of Type IIIA was 10–50% of that of Type IIIB, while the infection rate of Type HIC was 25–50%^4,5^. The medical costs per patient ranged from 17,000 to 150,000 US dollars^3^.

Severe multiple fractures have a higher likelihood of concomitant infection than single fractures, although the bacteriological culture of surrounding tissue or sinus secretions during surgery may yield negative results. However, some clinical manifestations similar to those associated with early infections, such as pain, fever, wound non-union or delayed healing, repeated swelling and pain, or continuous incision exudation, may still be observed preoperatively. Other clinical manifestations during the early stages of FRI mainly include wound redness and swelling, anorexia, fear of cold, chills, fever, vomiting, and so on. Some patients do not pay enough attention to the early changes in diseases or even forget the variations in clinical manifestations in the progression of diseases, making the diagnosis more difficult for clinicians^6,7^. When treating fractures, clinicians not only pay attention to the clinical characteristics of the patient, but also comprehensively consider other characteristics, such as imaging characteristics, microbiological features, and serum inflammatory markers.

FRI has always been a term difficult to define. Therefore, there are no accurate evaluation criteria, reference indicators, or diagnostic criteria for clinicians to assess the consequences of FRI. The serum inflammatory marker test is one of the most common auxiliary diagnostic methods for judging FRI in clinical practice. However, the specificity and sensitivity of these markers are not high. They are affected by the patient's age, gender, and other systemic comorbidities (such as tuberculosis, gout, tumors, and other diseases)^8–12^. Therefore, serum inflammatory markers cannot be used as the single basis for the differential diagnosis in clinical practice. Imaging examinations play a crucial role in the preoperative assessment of infectious bone nonunion. They can also be applied to the observation of post-fracture surgery changes in the anatomical structure, such as the presence of loose internal fixation structures or dislocation of fracture ends. Imaging examinations are preferentially employed for patients suspected of postoperative bone infection^8,13^. However, X-ray plain films, CT scans, or MRI (magnetic resonance imaging) are primarily used to evaluate the morphology, structure, and healing progress of fractures, with a limited ability in the diagnosis of infections^10,11,14^. In addition, the diagnostics based on CT and MRI often requires more time and higher expenses in China. The microbial culture method has been considered the "gold standard" for diagnosing FRI for a long period. It is widely used in clinics due to its large sample size, affordability, and convenience. However, this method has such limitations as low sensitivity and slow diagnosis, which can be mainly attributed to bacterial biofilm and other factors. In certain patients with anaerobic and caustic infections, specific culture techniques are required. False negative results may also occur, especially in infections with low virulence, special pathogens, or when antibiotics have been administered prior to sampling. The drawbacks of conventional microbiological culture methods become particularly evident in the identification of the etiology of FRI. Without proper guidance for etiological diagnosis, clinicians are left with no choice but to empirically administer broad-spectrum antibiotics, which often lead to ineffective treatment and poor prognoses^15–18^. However, the diagnosis of FRI involves a complex clinical process. Due to different affected sites and fracture types, as well as individual factors, there is still a lack of a single and universal diagnostic method to help clinicians make a definite diagnosis^17^.

The advancement of computer technology contributes to the emergence of various machine learning (ML) algorithms, such as SVM, Naive Bayes(NB), K-Nearest Neighbors(KNN), and RF, which have become increasingly prominent in various fields^20,21^. In the field of medicine, the availability of high-throughput data, characterized by large sample sizes and extensive features, has laid a solid foundation for the application of ML. This technology has been proven to be highly beneficial in clinical disease diagnosis, as it enables the analysis of vast amounts of patient data using algorithms and statistical models. Consequently, the accuracy of disease diagnosis is also significantly improved^22^. ML algorithms can be utilized to process and analyze large datasets containing patient information, such as medical records, genetic data, and clinical imaging results. These algorithms are designed to identify patterns and construct predictive models based on the data, allowing healthcare professionals to make more accurate and efficient diagnoses. However, ML algorithms rely on high-quality and comprehensive datasets for training and validation^23,24^. There may be challenges in obtaining and maintaining such datasets, especially in patients with FRI where data may be limited or incomplete. This study was conducted to establish a foundation for clinicians to formulate evidence-based and effective therapeutic regimens for patients with FRI. Specifically, the data of patients in the past 12 years were collected. Besides, a model was constructed based on the TSDPSO algorithm to optimize the SVM. Additionally, a multi-modal feature fusion method was employed to systematically analyze the data of patients with FRI. By utilizing this model and methodology, valuable insights and recommendations may be provided for clinicians to perform the diagnosis and treatment of patients with FRI.

The sample data were systematically retrieved, selected, and processed. In addition, ML algorithms were established, including SVM, RF, BP, XGBoost, and TSDPSO-SVM, under the guidance of four research questions (RQs). Due to the systematic nature of this study, it may be reproduced and updated in the future to reflect new activity. The four RQs are elucidated as follows.

1. How does the TSDPSO-SVM algorithm perform in identifying FRI?
2. What are the strengths and weaknesses of the TSDPSO-SVM algorithm in identifying FRI?
3. Does the age of individual patients affect the effectiveness of the TSDPSO-SVM algorithm in identifying FRI?
4. What are the potential applications and prospects of the TSDPSO-SVM algorithm in identifying FRI?

## Methods

A total of 7124 patients were enrolled in the Registration Office of Fracture-associated Infections in Qinhuai Medical Area of the Eastern Theater General Hospital of the Chinese People’s Liberation Army from 2010 to 2022. In this study, 4761 patients with long bone FRIs (aged 8–95 years, the FRI group) and 4761 patients with non-FRIs (aged 8–95 years, the Non-FRI group) were selected as study participants. The follow-up continued until August 2022. Besides, 97 features, including demographics, clinical features, laboratory test features, imaging features, and microbiological features, were extracted and collated from the Registration Office. All data used in this study were collected in accordance with relevant guidelines and regulations (Ministry of Science and Technology of the People's Republic of China, Policy No. 2006398). This study will be conducted in accordance with good clinical practice and ethical standards set out in the Declaration of Helsinki of 1964 and its subsequent amendments. As a retrospective study, only clinical data were collected from patients, without interfering with their treatment plans, and no physiological risks were posed to patients. In addition, informed consent was not obtained from participants due to objective reasons. The personal information related to all participants was protected properly. The need for informed consent was waived by the Ethics Committee of Jinling Hospital, owing to the retrospective nature of the study (Nanjing, China; 27 December 2022; approval number 2022NZKY-066-13).

### Support vector machine

SVM is a binary linear classifier. Identifying the decision boundary is an essential classification link, namely solving the hyperplane of the maximum margin of the learning sample^25,26^. The hyperplane equation can be expressed as:

1 $$f\left( x \right) = \omega^{T} x + b$$

where ω represents the normal vector of the hyperplane; B represents the offset of the hyperplane. By introducing the Lagrange coefficient, the objective function is transformed into a dual optimization problem:

2 $$\begin{gathered} \mathop {\min }\limits_{\omega ,b} \mathop {\max }\limits_{a} L\left( {\omega ,b,a} \right) = \frac{1}{2}\omega^{2} - \mathop \sum \limits_{i = 1}^{n} a_{i} \left[ {y_{i} \left( {\omega x_{i} + b} \right) - 1} \right] \left( {0 \le a_{i} \le c, i = 1,2,3, \ldots ,n} \right) \hfill \\ s.t.lossfunction: \mathop \sum \limits_{i = 1}^{n} a_{i} \left[ {y_{i} \left( {\omega x_{i} + b} \right) - 1} \right] \hfill \\ \end{gathered}$$

Lagrange coefficient c is a penalty factor. The penalty factor c affects the loss value of the objective function. The larger the c, the larger the error. When the error is large, the SVM is prone to over-fitting. When c is too small, SVM may have the under-fitting problem. In the linear SVM, the formula for solving the optimization problem is computed in the form of inner products. As the algorithm complexity of the inner product is very large, the kernel function is used to replace the inner product. In this study, the Gaussian radial basis kernel function was selected as the kernel function.

3 $$k\left( {x,x^{\prime } } \right) = \exp \left( { - \frac{{\left\| {x - x^{\prime } } \right\|^{2} }}{{2\sigma ^{2} }}} \right) = \exp \left( { - \gamma \left\| {x - x^{\prime } } \right\|^{2} } \right)$$

where $\sigma$ represents the radius of radial basis. The nuclear parameter $\gamma =\frac{1}{2{\sigma }^{2}}$ affects the training speed and test speed. In order to quickly identify the optimal parameters and improve the speed and accuracy of classification, the improved particle swarm optimization (PSO) was employed to optimize the SVM model in this study.

### Standard particle swarm optimization algorithm

Particle swarm optimization (PSO) is a swarm intelligence optimization algorithm proposed by Kennedy and Eberhart according to the predation behavior of birds^27^. The PSO algorithm involves a group of S particles moving at a certain speed in the D-dimensional search space, where the ith particle generates two vectors in the k-th iteration.

4 $$\left\{ {\begin{array}{*{20}c} {\nu_{i} \left( k \right) = \left[ {\nu_{i1} \left( k \right),\nu_{i2} \left( k \right),\nu_{i3} \left( k \right), \ldots ,\nu_{iD} \left( k \right)} \right]} \\ {x_{i} \left( k \right) = \left[ {x_{i1} \left( k \right),x_{i2} \left( k \right),x_{i3} \left( k \right), \ldots ,x_{iD} \left( k \right)} \right]} \\ \end{array} } \right.$$

The two vectors represent the “flying” speed and the vector position vector, respectively. During the iteration process, the position of each particle will be automatically adjusted to the global optimal direction, and the best position (pbest) established by each particle itself^28^. Pbest is expressed in ${{p}_{i}=(p}_{i1},{p}_{i2},{p}_{i3},\dots ,{p}_{iD})$. The best position in the whole group (gbest) is represented by ${{p}_{g}=(p}_{g1},{p}_{g2},{p}_{g3},\dots ,{p}_{gD})$. After the two best positions are identified, particles will update their speed and position according to Formulas (5) and (6).

5 $$\nu_{i} \left( {k + 1} \right) = \omega \upsilon_{i} \left( k \right) + c_{1} r_{1} \left\{ {p_{i} \left( k \right) - x_{i} \left( k \right) + c_{2} r_{2} \left[ {p_{g} \left( k \right) - x_{i} \left( k \right)} \right]} \right\}$$

6 $$x_{i} \left( {k + 1} \right) = x_{i} \left( k \right) + \nu_{i} \left( {k + 1} \right)$$

where $\omega$ represents the inertia weight; ${c}_{1}$ and ${c}_{2}$ represent acceleration coefficients; The two random numbers ${r}_{1}$ and ${r}_{2}$ are uniformly distributed in [0,1].

### Particle swarm optimization algorithm with traction switching delay

The PSO algorithm has a good optimization ability in solving the optimization function. Through iteration, the algorithm can quickly identify the optimal approximate solution, but it is easy to fall into local optimization and thus causes large errors^29^. In this study, a new traction switching delay particle swarm optimization (TSDPSO) was proposed based on the exchange particle swarm algorithm. The main idea of handover delay is to update the model adaptively according to the evolution factor and Markov chain^30^. Using the delay information of the optimal position of the particle itself and the optimal position of the particle population, the particle speed in the current iteration is updated according to the iteration state to eliminate local optimization and premature convergence of PSO. The speed and position update equations of the switching delay PSO algorithm are expressed in Formulas (7) and (8).

7 $$\nu_{ij} \left( {k + 1} \right) = \omega \left( k \right)\upsilon_{ij} \left( k \right) + c_{1} \left( {\xi \left( k \right)} \right)r_{1} \left\{ {\begin{array}{*{20}c} {p_{ij} \left[ {k - \tau_{1} \left( {\xi \left( k \right)} \right)} \right] - x_{ij} \left( k \right) + } \\ {c_{2} \left( {\xi \left( k \right)} \right)r_{2} \left\{ {p_{gj} \left[ {k - \tau_{2} \left( {\xi \left( k \right)} \right)} \right] - x_{ij} \left( k \right)} \right\}} \\ \end{array} } \right\}$$

8 $$x_{ij} \left( {k + 1} \right) = x_{ij} \left( k \right) + \nu_{ij} \left( {k + 1} \right)$$

where ${c}_{1}(\xi \left(k\right))$ and ${c}_{2}(\xi \left(k\right))$ represent the acceleration coefficients; ${\tau }_{1}(\xi \left(k\right))$ and ${\tau }_{2}(\xi \left(k\right))$ represent time delays. These four parameters are determined by the nonhomogeneous Markov chain $\xi \left(k\right) (k\ge 0)$. The values of Markov chain are s = {1, 2,…, n}. Its probability transfer matrix can be expressed as follows:

9 $$\begin{array}{*{20}c} {\begin{array}{*{20}c} {{\Pi }\left( k \right) = \left( {\pi_{ij} \left( k \right)} \right)} \\ {s.t. \mathop \sum \limits_{i = 1}^{n} \pi_{ij} \left( k \right) = 1 } \\ \end{array} } \\ {0 \le \pi_{ij} \left( k \right) \le 1} \\ \end{array}$$

In the TSDPSO algorithm, the probability transfer matrix $\Pi \left(k\right)$ is used for adaptive adjustment. According to the characteristics of the search process, four states can be defined according to the evolution factors: convergence, exploration, development, and jump out. These four states are respectively used in the Markov chain $\xi \left(k\right)=1, \xi \left(k\right)=2, \xi \left(k\right)=3 \mathrm{and} \xi \left(k\right)=4$. The average distance between each particle and other particles in the cluster can be expressed by ${d}_{i}$.

10 $$d_{i} = \frac{1}{s}\mathop \sum \limits_{j = 1}^{s} \sqrt {\mathop \sum \limits_{k = 1}^{D} \left[ {x_{i} \left( k \right) - x_{j} \left( k \right)} \right]^{2} }$$

where, s and D represent the size and size of particle swarm, respectively. The evolution factor $E_{f}$ is defined in Formula (11).

11 $$E_{f} = \frac{{d_{g} - d_{min} }}{{d_{max} - d_{min} }}$$

where $d_{g}$ represents the global best particle in the average distance di. $d_{max}$ and $d_{min}$ represent the maximum and minimum distances in di, respectively.

According to the value of $E_{f}$, the state of the Markov chain is determined by Formula (12).

12 $$\xi \left( k \right) = \left\{ {\begin{array}{*{20}c} {1, 0 \le E_{f} \le 0.25} \\ {2, 0.25 < E_{f} \le 0.5} \\ {\begin{array}{*{20}c} {3, 0.5 < E_{f} \le 0.75} \\ {4, 0.75 < E_{f} \le 1} \\ \end{array} } \\ \end{array} } \right.$$

Formula (9) can be modified as follows:

13 $${\Pi }\left( k \right)\left[ {\begin{array}{*{20}c} { \begin{array}{*{20}c} {x } & {1 - x} & { \begin{array}{*{20}c} {0 } & { 0 } \\ \end{array} } \\ \end{array} } \\ {\begin{array}{*{20}c} {\begin{array}{*{20}c} {\frac{1 - x}{2}} & { \begin{array}{*{20}c} {x } & {\frac{1 - x}{2}} & { 0 } \\ \end{array} } \\ \end{array} } \\ {\begin{array}{*{20}c} {\begin{array}{*{20}c} { 0 } & {\begin{array}{*{20}c} {\frac{1 - x}{2}} & { x } & {\frac{1 - x}{2}} \\ \end{array} } \\ \end{array} } \\ { \begin{array}{*{20}c} {0 } & { 0 } & {\begin{array}{*{20}c} {1 - x} & { x } \\ \end{array} } \\ \end{array} } \\ \end{array} } \\ \end{array} } \\ \end{array} } \right]$$

Therefore, the Markov process in the next iteration can switch its state based on the probability distribution matrix. In the iterative process, the inertia weight $\omega$ and the evolution factor $E_{f}$ have the same trend. A large $\omega$ tends to jump out and explore in the global search. Smaller $\omega$ is beneficial to local search. Assuming that the initial value of $\omega$ is set to 0.9, the function describing the inertia weight $\omega$ and $E_{f}$ is shown in Formula (14).

14 $$\omega \left( {E_{f} } \right) = 0.5E_{f} + 0.4$$

The initial values of acceleration coefficients $c_{1}$ and $c_{2}$ are set to 2, and they can automatically adjust their values according to the evolution state given in Table 1

**Table 1:** TSDPSO algorithm parameter setting.

State	$\xi \left(k\right)$	${c}_{1}$	${c}_{2}$
Convergence	1	2	2
Exploration	2	2.1	1.9
Development	3	2.2	1.8
Jump out	4	1.8	2.2

### Selection strategy of delay information

TSDPSO uses the delay information of pbest and gbest to update the velocity equation according to the evolution state. The strategy for selecting the delay information is elucidated as follows. In the jump state, the current globally optimal particle is willing to fly to a better solution, thus escaping from the local optimal solution. The delay information of pbest and gbest is more widely distributed in the search space. ${p}_{i}\left[k-{\tau }_{1}(\xi \left(k\right))\right]$ and ${p}_{g}[k-{\tau }_{2}(\xi \left(k\right))]$ are the best locations of the particles and groups encountered in the last iteration, which contain information about particles and groups. Therefore, selecting them to update the velocity equation contributes to jumping out of the local optimum. In the exploration state, selecting the delay value of pbest and gbest in the current iteration allows the particles to make autonomous exploration and also guides them to the historical global best position. In the development state, each particle uses its historical best position in the current iteration ${p}_{i}\left[k-{\tau }_{1}(\xi \left(k\right))\right]$ and gbest, which can enhance local search and development. In the convergence state, all particles are willing to converge to the optimal solution as soon as possible within the global optimal region found. Therefore, particles should follow pbest and gbest in the current iteration to achieve the goal in this state.

### Traction operation

**Figure 1:**
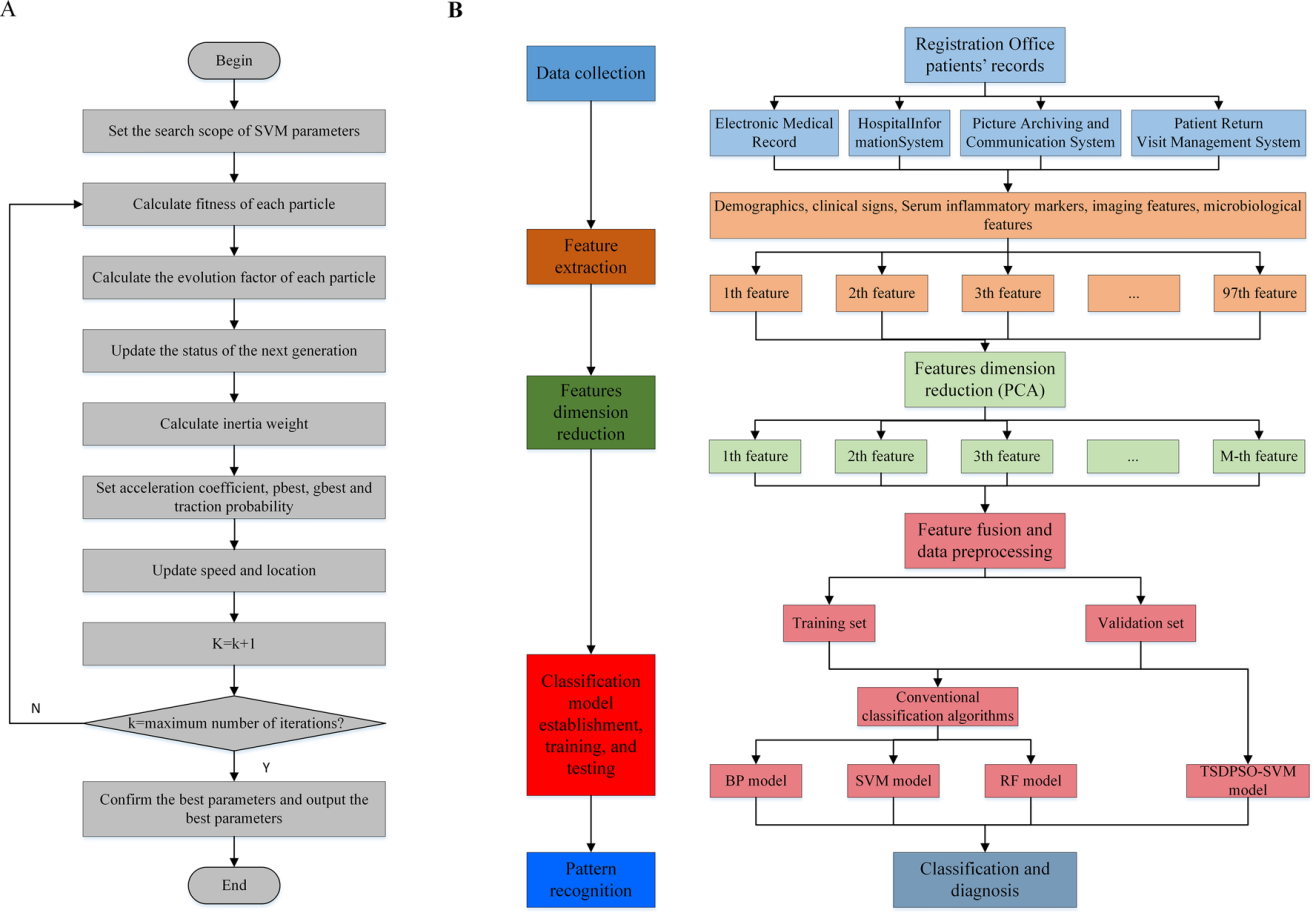
Flowchart of data collection and algorithm optimization process in this study. (**A**) The Process of TSDPSO_SVM. (**B**) The overall design process.

Traction operation (PO) can be used to perform particle traction operation on the best position direction of the trapped particle in the last 10 movements in order to accelerate the convergence speed of PSO when the particle falls into the search area with poor fitness values^31^. The operation allows particles to quickly leave the global poor region and search for the global optimal solution region, which can improve the particle search speed and algorithm convergence speed.

15 $$PO = \frac{{f_{i} - f_{min} }}{{f_{avg} - f_{min} }}rand\left( 1 \right)\left\{ {p_{g} \left[ {k - \tau_{1} \left( {\xi \left( k \right)} \right)} \right] - p_{i} \left[ {k - \tau_{1} \left( {\xi \left( k \right)} \right)} \right]} \right\}$$

where $f_{i}$ represents the fitness value of particle i; $f_{min}$ represents the minimum fitness value of the population; $f_{avg}$ represents the average fitness value of the population. The adaptive traction factor $\frac{{f_{i} - f_{min} }}{{f_{avg} - f_{min} }}$ exerts a significant impact on the particles in the poor search area. In this study, PO was used to update the speed and position of particles (Formulae (7) and (8)). Figure 1A presents the TSDPSO algorithm to optimize SVM parameters.

### Main reagents and instruments

A 128 row CT machine (Siemens, Germany), full-automatic biochemical analyzer (Sumikon, Japan), high-speed centrifuge (Shanghai Luxianyi Centrifuge Instrument Co., Ltd.), ultraviolet spectrophotometer (Hitachi Scientific Instrument Co., Ltd., USA), full-automatic radioimmunoassay γ counter (Science and Technology University Innovation Co., Ltd.), VITEK 2 compact full-automatic microbial analysis system (France BioMerier Co., Ltd.), API qualification system (France BioMerier Co., Ltd.), and Autopol V Plus AutoFill (Rudolph Company, USA) were adopted in this study.

### Data processing

Data preprocessing is one of the biggest challenges in data mining. Typically, data preprocessing includes identification and processing of non-normal data, data coding, missing value process, data transformation and integration, data dimension reduction, etc. The uncertainty, inconsistency, and missing values of data are also other challenges in the data mining process. In this study, the collected data were preprocessed through the following steps^32–35^.

#### Data cleaning

The primary objective of data cleaning is to ensure the accuracy, completeness, and reliability of the data for subsequent analysis. Firstly, the data inspection stage entails carefully examining the data for any evident mistakes or discrepancies, such as missing values, duplicate entries, or incorrect data formats. Through the inspection process, a deeper understanding of the organization and potential problems related to datasets can be obtained. Then, any missing or duplicate data are eliminated from the collected dataset to ensure the integrity and uniqueness of the data. Moreover, the data are converted into a common data type that can be easily processed and analyzed. For example, if there is a mix of numeric and string data, all the values can be converted into numeric data if possible. Furthermore, in statistics, an outlier is an observation that lies an abnormal distance from other values in a random sample from a population. These extreme values can distort the results of the analysis and hence should be either removed or adjusted. In this study, the Z-score statistical method was employed to detect outliers ($68.26\%\in [-\sigma ,\sigma ])$. Once identified, the rows that contain these outliers would be excluded from the dataset.

#### Feature normalization

Feature normalization is a technique used in ML and data analysis to standardize the features or variables of a dataset. It involves transforming the values of features so that they can have a similar scale or range. The purpose of feature normalization is to ensure that no specific feature dominates the learning process or algorithm due to its larger value range. This contributes to improving the performance and accuracy of the model. In this study, a method called min–max normalization was used to scale the features to a specific range. This normalization technique can be utilized to standardize the features and make them more comparable.

16 $$\hat{x}_{i} = \frac{{x_{i} - \mathop {\min }\limits_{j = 1 \ldots n} x_{j} }}{{\mathop {\max }\limits_{j = 1 \ldots n} x_{j} - \mathop {\min }\limits_{j = 1 \ldots n} x_{j} }}$$

#### Data reduction

In datasets containing a large number of variables, dimensionality reduction techniques can be employed to decrease the number of variables while preserving the most significant information. The Principal Component Analysis (PCA) is a widely used method for dimensionality reduction in data analysis. As it can identify the most crucial features and simplify the dataset, this method exhibits special advantages in dealing with high-dimensional data. The PCA algorithm comprises several steps, which are outlined below:

Step 1: Standardization: The first step in PCA is to standardize the data using the min–max method, where the minimum value is set to 0 and the maximum value is set to 1. This can be achieved by subtracting the mean from each feature and dividing by the standard deviation. Standardization ensures that all features are on the same scale, which is necessary for PCA to function effectively.

Step 2: Calculation of the Covariance Matrix: After standardization, the next step involves the calculation of the covariance matrix. The covariance matrix quantifies the relationship between each pair of features. It is a square matrix, with each element representing the covariance between two features. The covariance between feature i and feature j is calculated as the average of the product of their standardized values.

17 $$C = \frac{1}{n}{*}XX^{T}$$

The formula above represents the covariance matrix C. In this formula, n represents the sample size, X represents the standardized data matrix, and ${X}^{T}$ represents the transpose of X.

*Step 3: Eigenvalue decomposition* Once the covariance matrix is calculated, it would be decomposed into corresponding eigenvectors and eigenvalues. The eigenvectors represent the directions in the original feature space where the data varies the most, while the eigenvalues represent the amount of variance explained by each eigenvector. These eigenvectors are also referred to as the principal components.

*Step 4: Selection of principal components* In this step, the principal components can be selected based on their corresponding eigenvalues, with the aim of selecting the principal components that can explain the most variance in the data. These principal components can be ranked in descending order using the eigenvalues. A threshold can be set to determine the number of principal components to retain. To find the optimal eigenvectors, the scale parameter (n_components) can be set within the range of [0,1].

*Step 5: Projection* After selecting the principal components, the data can be projected onto the new feature space defined by these components. This can be achieved by taking the dot product of standardized data and selected principal components. The result is a transformed dataset with reduced dimensionality.

SPSS24.0 was used to randomly select 80% of the samples as the training set (3809 patients in the FRI group and 3809 patients in the Non-FRI group) for the training of the model, and 20% of the samples as the test set (1904 patients in the FRI group and 1904 patients in the Non-FRI group). The quantitative characteristic values (height, age, etc.) of samples were normalized, so that the sample characteristics were distributed between [− 1,1]. This can eliminate the impact of differences in the quantity order and unit of quantitative samples on model training and prediction. The feature set of initially selected patients comprised medical imaging features, demographic features, clinical signs, microbiology features, and serum inflammatory markers. In this study, the PCA was adopted to reduce the dimension of features^36^.

### Model establishment and evaluation

A multi-dimensional feature fusion joint classification algorithm was proposed to design an FRI recognition method. The overall design process of this method is shown in Fig. 1B. After feature extraction, feature dimensionality reduction, and feature fusion, the training set and test set of patients in the FRI group and benign group were trained and tested, respectively, by SVM, BP, RF, XGBoost, and TSDPSO-SVM. Evaluation indicators, including the confusion matrix, accuracy, area under the curve (AUC), recall (TPR, sensitivity), specificity, F1, false positive rate (FPR,1- specificity), and precision calculated by true positives (TP), false positives (FP), true negatives (TN), and false negatives (FN), were used to evaluate the performance of each model. A higher AUC value indicated a better overall performance of the current feature. AUC < 0.5 indicated that there was no diagnostic significance; AUC ranging from 0.5 to 0.7 indicated that the diagnostic accuracy of the model was low; AUC ranging from 0.7 to 0.9 indicated that the degree of authenticity was good; AUC > 0.9 indicated that the degree of authenticity was very high.

### Ethics approval and consent to participate

As a retrospective study, the research involving human subjects underwent review and received approval from the Ethics Committee of Jinling Hospital. The study adhered to the guidelines set in the Helsinki Declaration. The need for Informed Consent was waived by the Ethics Committee of Jinling Hospital, owing to the retrospective nature of the study (Nanjing, China; 27 December 2022; approval number 2022NZKY-066-13).

## Results

### Simulation analysis of algorithm performance

In order to verify the performance of TSDPSO, six common algorithm performance test functions (Griewank function, Rastigin function, Alpine function, Ackley function, Rosenbrock function, and Sphere function) were selected to evaluate the algorithm performance. The six functions can be expressed as follows:

$$\begin{array}{*{20}l} {{\text{Griewank}}:} \hfill & {f\left( {\text{x}} \right) = 1 + \frac{1}{4000}\mathop \sum \limits_{i = 1}^{n} x_{i}^{2} - \prod\limits_{i = 1}^{n} {\cos \frac{{x_{i} }}{\sqrt i }} } \hfill \\ {Rastigin:} \hfill & {f\left( x \right) = 10n + \mathop \sum \limits_{i = 1}^{n} \left[ {x_{i}^{2} - 10cos\left( {2\pi x_{i} } \right)} \right]} \hfill \\ {Alpine:} \hfill & {f\left( x \right) = \sum\limits_{i = 1}^{n} {\left| {x_{i} \sin \left( {x_{i} } \right) + 0.1x_{i} } \right|} } \hfill \\ {{\text{Ackley}}:} \hfill & {{\text{f}}\left( {\text{x}} \right) = - 20{\text{exp}}\left( { - 0.2\sqrt{\frac{1}{n}} \mathop \sum \limits_{i = 1}^{n} x_{i}^{2} } \right) - {\text{exp}}\left( {\frac{1}{n}\mathop \sum \limits_{i = 1}^{n} \cos \left( {\pi i} \right)} \right) + 20 + e} \hfill \\ {Rosenbrock:} \hfill & {f\left( x \right) = \mathop \sum \limits_{i = 1}^{n - 1} \left[ {100\left( {x_{i + 1} - x_{i} } \right)^{2} + \left( {x_{i} - 1} \right)^{2} } \right]} \hfill \\ {Sphere:} \hfill & {f\left( x \right) = \mathop \sum \limits_{i = 1}^{n} x_{i}^{2} } \hfill \\ \end{array}$$

**Figure 2:**
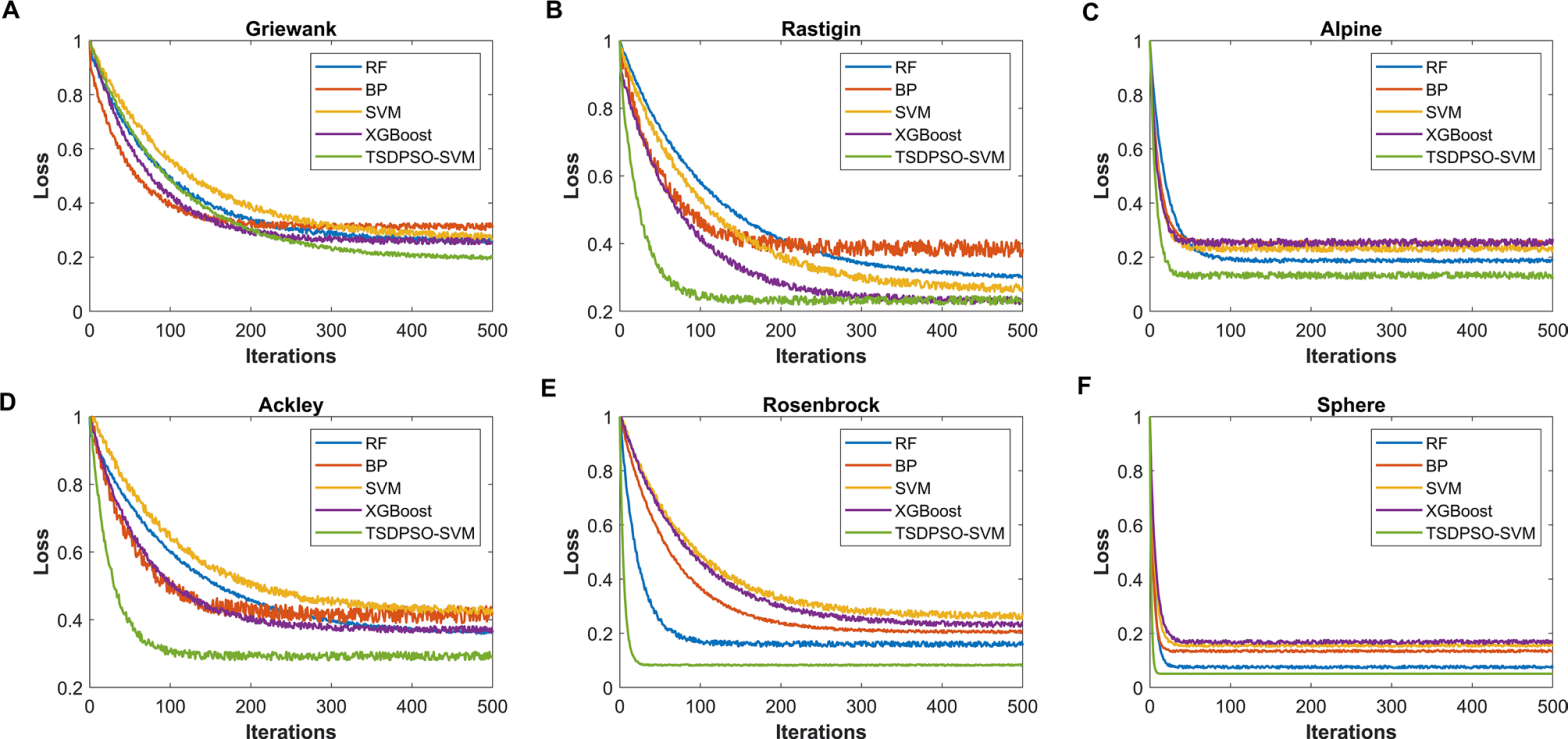
Performance test results of these algorithms. (**A**) Results of the algorithm performance test using Griewank function (**A**), Rastigin function (**B**), Alpine function (**C**), Ackley function (**D**), Rosenbrock function (**E**), and Sphere function (**F**). The abscissa represents the number of iterations, and the ordinate represents the missing function.

In this study, six test functions were selected to test the performance of TSDPSO-SVM (Fig. 2). The superiority of TSDPSO-SVM was verified by comparing the results of six test functions of the BP neural network model, RF random forest model, SVM model, and XGBoost model. Through comparison, it was found that compared with the other three algorithms, TSDPSO-SVM delivered the best robustness, the fastest convergence speed, and the best optimization ability.

### Results of feature dimension reduction based on the PCA method

**Figure 3:**
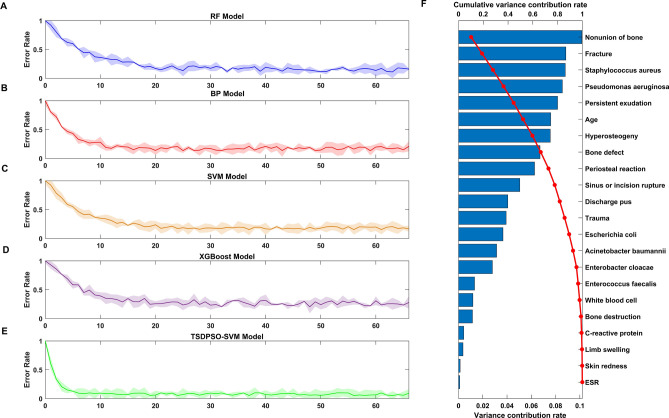
Results of feature dimension reduction based on the PCA method. RF (**A**), BP (**B**), SVM (**C**), XGBoost (**D**), and TSDPSO-SVM (**E**). When setting the parameters for the RF model, there are several important factors to consider: Number of trees: 100, Maximum depth: 6, Minimum samples split: 2, Minimum samples per leaf: 1, Maximum features: 5. The parameters of the BP model can exert a significant impact on its performance and convergence. There are several important parameters that need to be set: Learning rate (η): 0.01, Activation function: sigmoid, Training target error: 1e−3, Maximum number of iterations: 10,000, Power factor: 0.9, and Number of hidden layers and neurons: 2 and 10*6. When training an SVM model, there are several important parameters that need to be considered: Kernel function: RBF, Penalty parameter: 1, and Error convergence conditions: 1e−3, and there is no limit on the maximum number of iterations. Some important parameter settings for the XGBoost model are presented as follows: Booster type: gbtree, Learning rate: 0.1, Gamma: 0, Lambda: 0, Alpha: 0, Maximum depth of a tree: 6, Minimum sum of instance weight needed in a child: 1, Subsample ratio of the training instance: 1, Subsample ratio of columns when constructing each tree: 1, and Number of boosting rounds: 10. In the TSDPSO-SVM model, there are several important parameters that need to be set: Swarm size: 20, Maximum number of iterations: 500, Cognitive parameter (C1): 2, Social parameter (C2): 2, Maximum speed: 4, Inertia weight: 0.9, and Convergence criteria: 1e−3. All parameter settings in the SVM process are specific to the SVM model. In the models, the feature set after dimensionality reduction is used to conduct unsupervised learning on FRI case samples. The abscissa represents the number of features in the feature set. The ordinate represents the test error rate. (**F**) Results of the variance contribution rate and cumulative variance contribution rate of the “optimal feature set”. The histogram represents the variance contribution rate, and the red line represents the cumulative variance contribution rate. The ordinate represents the selected feature set.

The multi-dimensional feature data set collected in this study included 97 features, covering medical imaging features, demographic features, clinical signs, microbiology features, and serum inflammatory markers of patients. In this study, the PCA method was employed to reduce the dimensions of these collected multidimensional features. In order to find the optimal eigenvector, the scale parameter (n_components) was set to be [0,1] and dynamically increased. BP, RF, SVM, XGBoost, and TSDPSO-SVM were used to perform unsupervised learning on the “optimal feature set” after dimensionality reduction. The results are shown in Figs. 3A–E. When the value of n_components was 0.227, the training accuracy of each model tended to be stable, and the number of features after dimensionality reduction was 22. When the value of n_components was 0.227, the variance contribution rate and cumulative variance contribution rate were analyzed for the selected “optimal feature set” (Fig. 3F).

### Identification results of each model

**Figure 4:**
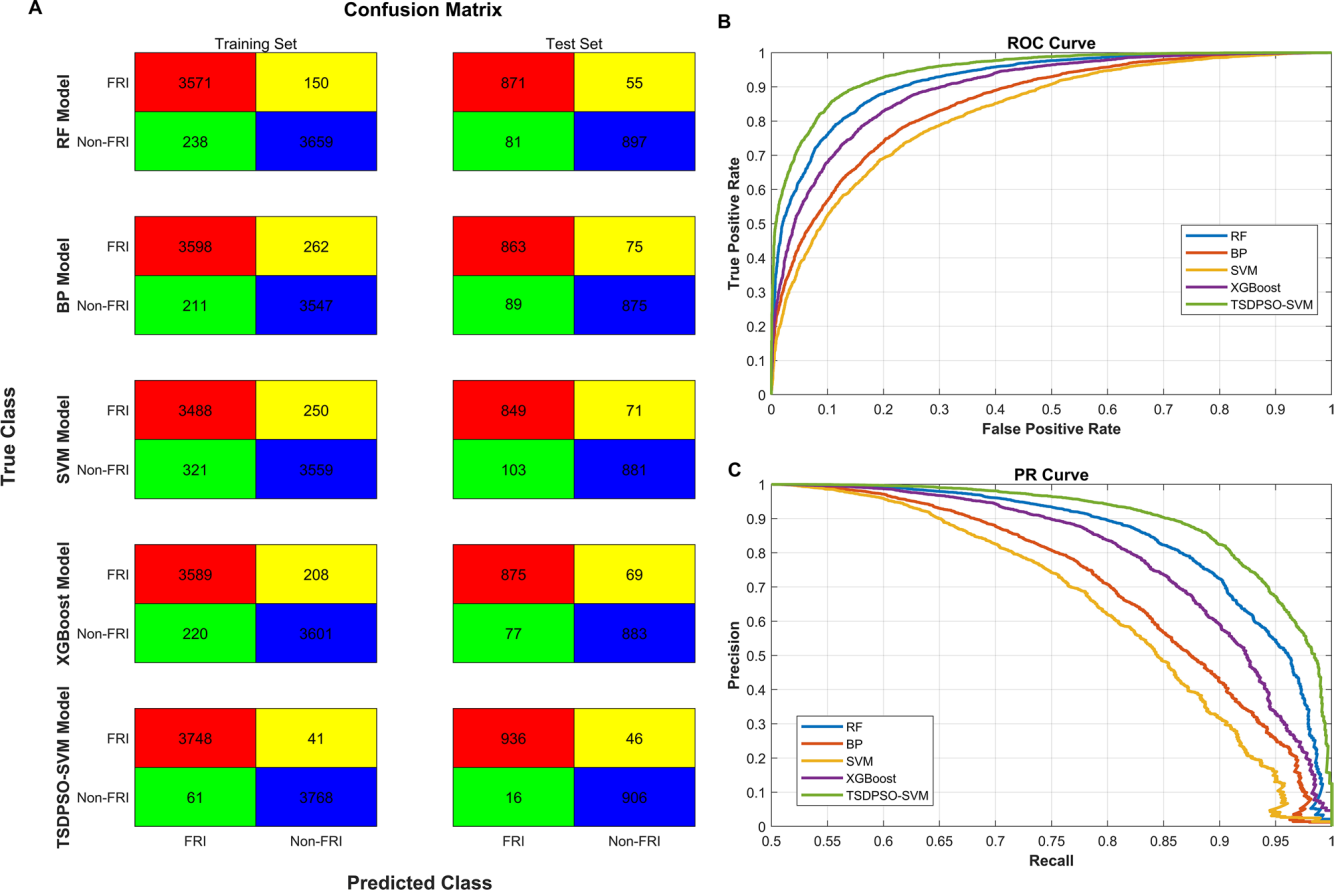
Calculation results of training and test accuracy of each model. (**A**) The recognition results of five models in the training set, in which red represents TP, green represents FN, yellow represents FP, and blue represents TN. (**B**) ROC curves of five models. (**C**) PR curves of five models.

According to PCA, 22 indicators were selected, including nonunion of bone, fracture, staphylocus aureus, pseudomonas aeruginosa, persistent exudation, age, hyperosteogeny, bone defect, periodic reaction, sinus, fibrous or decision rule, discharge pus, trauma, escherichia coli, etc., to establish models, including RF, BP, SVM, XGBoost, and TSDPSO-SVM. First, the data were preprocessed as described above and then randomly divided into a training set (7618, 80%) and a test set (1904, 20%). The proportion (1:1) of the FRI group and the Non-FRI group in the training set was consistent with that in the test set. Besides, the tenfold cross-test method was utilized to train the model and obtain the best model parameters. Specifically, the training set was divided into ten parts, nine of which were used to train the model, and the rest one was used to validate the model. The average value of ROC (AV-ROC) (calculated ten times) was recorded as an indicator of the evaluation model. The number of true positive (TP), false positive (FP), true negative (TN), and false negative (FN) of the training model and test model results were recorded. The evaluation indicators of each model, including recall, specificity, F1 score accuracy, area under the curve (AUC), recall (TPR, sensitivity), specificity, F1, false positive rate FPR, and specificity, were calculated by TP, FP, TN, and FN. The training and test results are shown in Fig. 4A. The test results are shown in Table 2. The ROC curve and PR curve of the five models are plotted, as shown in Figs. 4B and 4C.

**Table 2:** Test results of each model in the test set. AUC represents the area under the curve value of the variable.

Model	AUC	ACC (%)	Recall (%)	Specificity (%)	FPR (%)	Precision (%)	F1
RF	0.86	92.86	91.49	94.22	5.78	94.06	0.93
BP	0.79	91.28	90.65	92.11	7.89	92.00	0.91
SVM	0.74	90.86	89.18	92.54	7.46	92.28	0.91
XGBoost	0.84	92.33	91.91	92.75	7.25	92.69	0.92
TSDPSO-SVM	0.91	96.74	98.32	95.17	4.83	95.32	0.97

**Figure 5:**
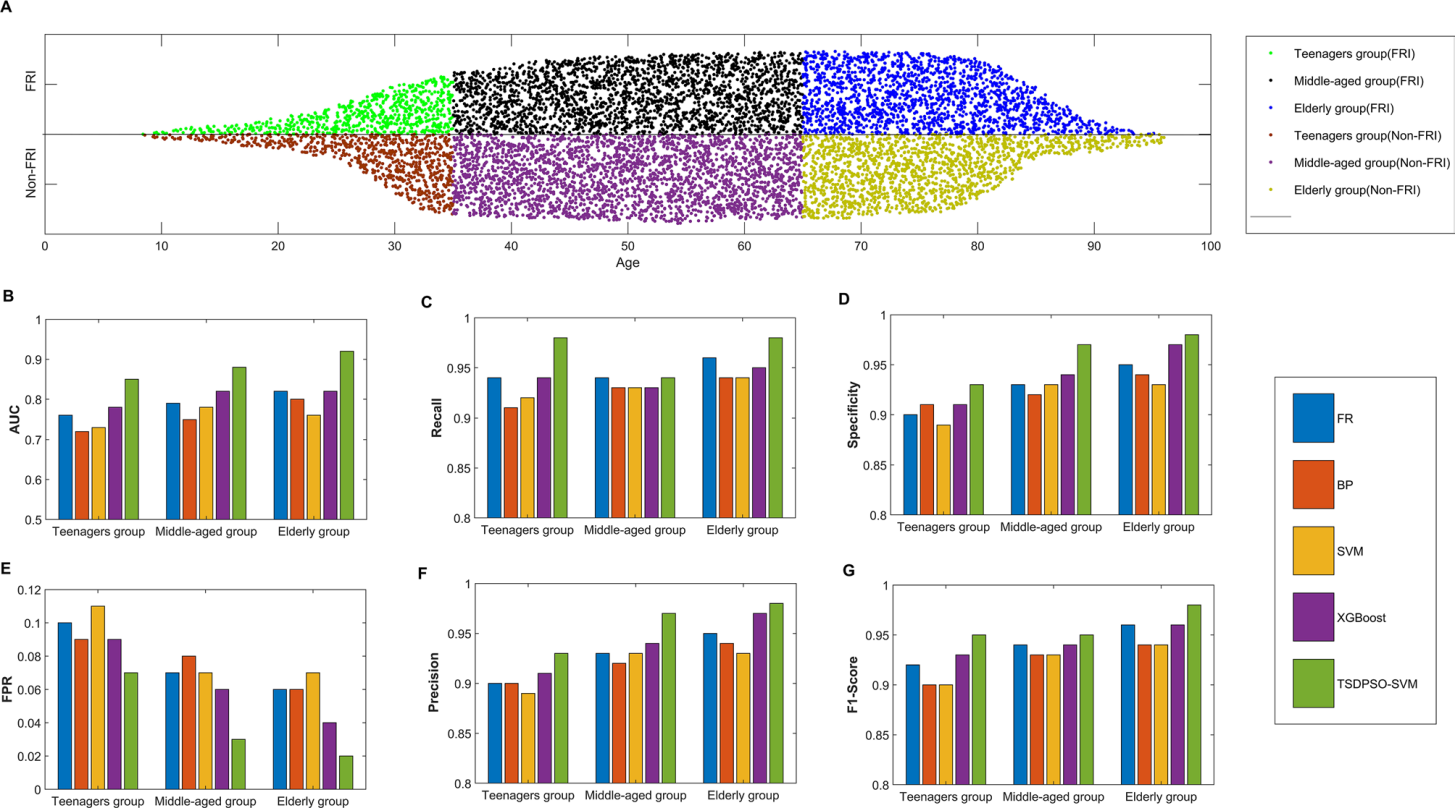
Age distribution of patients in each group and test results of patients in each group. (**A**) Age distribution results of FRI and Non-FRI patients. Green represents the age distribution of adolescent patients with FRI, comprising 864 patients. Black represents the age distribution of middle-aged patients with FRI, comprising 1766 patients. Blue represents the age distribution of the elderly with FRI, comprising 2131 patients. Additionally, brown represents a group of adolescents with Non-FRI, consisting of 921 patients. Purple represents middle-aged patients with Non-FRI, totaling 1842 patients. Lastly, yellow represents the elderly with non-FRI, including 1998 patients. (**B**–**G**) represent the evaluation metrics of the test set for each age group used by RF, BP, SVM, XGBoost, and TSDPSO-SVM. These metrics include the area under the curve (AUC), recall, specificity, false positive rate (FPR), precision, and F1 score.

As per these findings, age was identified as a significant factor. However, clinical observations suggested that there might be variations in infection characteristics among patients in different age groups. As a result, for the purpose of this study, patients were divided into four distinct groups based on their age: the children group (8–17 years old, FRI vs. Non-FRI: 64 vs. 107), the youth group (18–34 years old, FRI vs. Non-FRI: 800 vs. 814), the middle-age group (35–65 years old, FRI vs. Non-FRI: 1,766 vs. 1842), and the elderly group (≥ 66 years old, FRI vs Non-FRI: 2131 vs. 1998). To ensure statistical validity, the children group and the youth group were combined into a single teenager group due to the limited number of patients. The classification is illustrated in Fig. 5A.

In this study, five models (RF, BP, SVM, XGBoost, and TSDPSO-SVM) were constructed based on other 21 features, including nonunion of bone, fracture, staphylocus aureus, pseudomonas aeruginosa, etc. (without age). Each age group underwent separate analyses, and five models were also trained based on these patients, with 80% of patients as the training set and the remaining 20% as the test set. The results of these analyses are presented in Table 3 and Figs. 5B–G.

**Table 3:** Calculation results of training and test accuracy of each age group.

Age group	Model	Group	Training set		Acc of training set (%)	Test set		Acc test set (%)
			FRI	Non-FRI		FRI	Non-FRI	
Teenagers group	RF	FRI	644	59	92.58	162	18	91.88
		Non-FRI	47	678		11	166	
	BP	FRI	631	68	91.04	157	17	90.76
		Non-FRI	60	669		16	167	
	SVM	FRI	634	66	91.39	159	20	90.48
		Non-FRI	57	671		14	164	
	XGBoost	FRI	646	49	93.42	163	16	92.72
		Non-FRI	45	688		10	168	
	TSDPSO-SVM	FRI	659	24	96.08	169	12	95.52
		Non-FRI	32	713		4	172	
Middle-aged group	RF	FRI	1334	103	93.70	333	26	93.62
		Non-FRI	79	1371		20	342	
	BP	FRI	1331	110	93.35	329	29	92.65
		Non-FRI	82	1364		24	339	
	SVM	FRI	1328	123	92.80	328	24	93.20
		Non-FRI	85	1351		25	344	
	XGBoost	FRI	1358	98	94.70	329	21	93.76
		Non-FRI	55	1376		24	347	
	TSDPSO-SVM	FRI	1389	53	97.33	332	12	95.42
		Non-FRI	24	1421		21	356	
Elderly group	RF	FRI	1644	77	95.82	411	22	95.52
		Non-FRI	61	1521		15	378	
	BP	FRI	1619	94	94.55	400	25	93.83
		Non-FRI	86	1504		26	375	
	SVM	FRI	1611	97	94.22	401	29	93.46
		Non-FRI	94	1501		25	371	
	XGBoost	FRI	1631	57	96.03	406	14	95.88
		Non-FRI	74	1541		20	386	
	TSDPSO-SVM	FRI	1698	30	98.88	418	9	97.94
		Non-FRI	7	1568		8	391	

## Discussion

In the twenty-first century, with the advancement of transportation and industry, the incidence of various high-energy injuries caused by traffic accidents and high-altitude falls has increased significantly^37,38^. Most of these patients have open wounds, with a significantly increased risk of soft tissue injury and exposure to external conditions. The risk of postoperative infection is also increased. In recent years, although many surgical treatments have been applied in clinical practice, surgical stability and anti-infection treatment have reached a certain level. The incidence of postoperative infection is still high, and perioperative anti-infection treatment remains a clinical challenge^39^. FRI is a common serious complication in orthopedics. Staphylococcus aureus and Escherichia coli are the main pathogens of FRI. It has been demonstrated that FRI will not only aggravate the patient's condition but also increase the difficulty of treatment^40^. Postoperative infection without timely treatment can easily affect the efficacy of orthopedic surgery, and may even induce lung infection, kidney infection, and other life-threatening diseases. Therefore, in the treatment of such patients, it is of great significance to predict the FRI of patients by analyzing the infectivity index of patients in time to improve the prognosis of patients^41^.

The lack of a clear definition has hindered the diagnosis and treatment of FRI. According to the research group of 2018 International Consensus Conference, suppurative drainage and wound dehiscence/rupture are considered confirmatory signs of infection, while other local clinical symptoms, such as pain, fever, redness, and swelling, can only be regarded as indicative features of infection^42,43^. It is important to interpret serum inflammatory markers carefully when diagnosing FRI. Although patients with FRI tend to exhibit higher levels of ESR, CRP, and lysozyme activity, the diagnosis of FRI cannot be made relying solely on a single serum inflammatory marker^44^. Common imaging methods used for diagnosing FRI include conventional X-ray, CT, and MRI. In addition, techniques such as bone scan (BS), positron emission tomography (PET), and scintigraphy using white blood cells or antigranulocyte antibodies are also employed^45–47^. Typically, conventional radiography is the initial step when FRI is suspected. Radiological signs that suggest FRI include implant loosening, bone lysis, non-union, sequestration, and periosteal bone formation. The choice of imaging modality depends on such factors as local availability, clinical inquiries, and the expertise of medical specialists^48^. Although nuclear imaging has favorable diagnostic accuracy, the presence of FRI cannot be definitively established only based on this method. While conventional culture is considered the “gold standard” for diagnosing bacterial infections, it may still produce false negative and false positive results for complex fractures like tibial plateau fractures^49,50^.

In recent years, there has been an increasing number of scholars conducting research on the diagnosis and treatment of FRI, which has resulted in significant advancements. In 2019, Justin V C Lemans et al. conducted a retrospective cohort study that included all patients who were suspected of having FRI and underwent 18F-FDG PET/CT at two primary trauma centers between 2011 and 2017. The qualitative assessment of 18F-FDG PET/CT scans showed a sensitivity of 0.89, specificity of 0.80, PPV of 0.74, NPV of 0.91, and diagnostic accuracy of 0.83^14^. In 2022, Hassan Farooq et al. conducted a prospective case–control study to compare plasma protein inflammatory biomarkers and mid-infrared (MIR) spectral patterns between patients with confirmed FRI and an uninfected control group. They developed a predictive model based on multivariate analysis and enzyme-linked immunosorbent assay–based biomarkers. The model had a sensitivity of 69.2% and an accuracy of 84.5%^12^. In 2018, a quantitative study was conducted by M. Morgenstern et al. This study aimed to analyze the clinical features, microbiology culture results, and historical data of 156 ecologically treated non-observations in order to determine the likelihood of associated infections. A cut-off point was established, whereby the absence of neutrophils in any high-power field was used to diagnose aseptic non-union. The study found a sensitivity of 85% and specificity of 98%, resulting in an overall accuracy of 92%^15^.

In this study, the records of 4761 patients from the FRI Registry of Qinhuai Medical District, the General Hospital of the Eastern Theater of the Chinese People’s Liberation Army from January 1, 2010 to May 31, 2022 were retrospectively analyzed. Besides, multidimensional characteristics of the sample set were collected, including imaging features, demographic features, clinical signs, microbiology features, and serum inflammatory markers. In addition, the principal component analysis (PCA) method was used to reduce the dimension of features, thus reducing the redundancy of data and improving the generalization ability of the models. A new classification model was developed based on the improved PSO SVM model. Guided by the selection strategy and traction operation of delayed information, the particles can jump out of the local optimum and converge to the global optimum faster. Moreover, five ML algorithms, including TSDPSO-SVM, were used to distinguish FRI from Non-FRI. A 4761 * 2 * 22 data set was used and randomly divided into the training set and the test set with a ratio of 8:2 (FRI: Non-FRI = 1:1).

In this study, the data on patients with FRI over the past 12 years were collected, and a novel ML algorithm called TSDPSO-SVM was also constructed. Additionally, RF, BP, SVM, and XGBoost models were also established based on the research objectives and research questions (RQs). Through these scientific efforts, the following conclusions regarding the four RQs were reached.

*RQ-1* In order to evaluate the performance of the TSDPSO-SVM algorithm in identifying FRI, a series of experiments were conducted. Compared with conventional diagnostic methods, TSDPSO-SVM increased the accuracy in the recognition of FRI by 4.74–13.74%^12,14,15^. By comparing the accuracy, recall, precision, and F1 score of TSDPSO-SVM with those of RF, BP, SVM, and XGBoost, its effectiveness can be verified. Griewank and other test functions were also adopted to validate the superiority of TSDPSO-SVM. FRI and Non-FRI were distinguished by using the sample characteristics collected from various aspects. The AUC of TSDPSO-SVM was higher than 0.91, at least 5% higher than that of the other three models. Compared with Random Forest (RF), Backpropagation Neural Network (BP), and SVM, TSDPSO-SVM demonstrated remarkable accuracy in the test set (${\chi }^{ 2}=29.17, 50.46, \mathrm{56.66,35.88}, P<0.01$). The recall of TSDPSO-SVM was also significantly improved (${\upchi }^{2}=91.78, 107.42, 135.69, 97.61\mathrm{P}<0.01$). Compared with BP and SVM, the specificity of TSDPSO-SVM was significantly improved (FPR was significantly reduced) (${\chi }^{2}=14.84, 11.38, P<0.01$). In terms of precision, the same results were obtained in TSDPSO-SVM. Compared with other algorithms, TSDPSO-SVM has the best robustness, the fastest convergence speed, and the best optimization ability.

*RQ-2* Regarding the identification of FRI, TSDPSO-SVM may have the following advantages. SVM performed well in handling non-linear classification problems, and TSDPSO can help optimize the parameters of SVM models, thereby improving accuracy and stability (They have been described in RQ-1). However, TSDPSO-SVM also has some limitations. For example, it has higher computational complexity and lacks interpretability, which can be considered as black boxes, indicating that it is difficult to explain the decision-making process. This limitation may cause a hindrance to clinicians to understand and trust the recommendations made by these algorithms.

*RQ-3* These patients were also divided based on their age, and five models were also utilized to distinguish FRI from non-FRI patients. Besides, it was also confirmed that the age of individual patients can significantly affect the effectiveness of TSDPSO-SVM in identifying infections. This implied that fracture risks associated with different age groups may vary, and hence age-specific factors should also be considered. Through multiple experiments, it was observed that although TSDPSO-SVM may exhibit slight fluctuations in accuracy, the recall, accuracy, and F1 score can remain consistently high across various age groups. For instance, TSDPSO-SVM consistently achieved a recognition accuracy above 95% and an accuracy exceeding 93% for different age groups. These findings demonstrated the robustness of TSDPSO-SVM in accurately distinguishing between FRI and Non-FRI patients across diverse age groups.

*RQ-4* TSDPSO-SVM was validated to be stable across various sample sizes, age compositions, and data distribution patterns. This contributed to its high applicability in scenarios involving the detection of FRI. In the field of clinical medicine, this algorithm can aid doctors in accurately assessing the risk of infections in patients, leading to favorable treatment strategies. Furthermore, the versatility of this algorithm implies its potential application in such fields as biomedical research and predictive modeling.

In this study, the multiple eigenvector fusion technology was used to evaluate and compare the performance of five models in the differentiation between FRI and Non-FRI. It was validated that TSDPSO-SVM had the maximum overall prediction ability and could effectively distinguish between FRI and Non-FRI. In addition, 22 characteristics, such as nonunion of bone, fracture, Staphylococcus aureus, Pseudomonas aeruginosa, persistent exudation, and age, were ranked based on their medical importance in the differentiation between FRI and Non-FRI. These efforts may also conduce to the early diagnosis of FRI and can also provide reference for clinicians, especially those with insufficient experience. Further, it was found that the cumulative interpretable variation of the top 9 features exceeded 80%, which may exert a greater impact on the predictability of the model. Despite certain accomplishments in this study, there are still some limitations and assumptions that need to be addressed.

1. *Limitations of data collection* Data collection of FRI is a complex and labor-intensive task. In this study, it can be assumed that accurate and comprehensive datasets related to FRI have been collected. However, in the real world, due to many limitations, such as limited time, resources, and manpower, there may be a failure to obtain datasets with sufficient scales and diversities.
2. *Limitations of feature selection* In this study, a multi-dimensional feature fusion method was adopted to combine information from different feature dimensions for the identification of FRI. However, in practical applications, there may be a failure to obtain all potentially valuable features. Additionally, there may also be deviations or errors in feature selection, which could result in a decrease in the model's performance.
3. *General limitations of experimental results* This study primarily focuses on the specific issue of FRI, and hence the model's performance can only be evaluated in this particular context. However, the application and performance of this model in other medical fields still require further evaluation and validation.
4. *Limitations on interpretability* As a black box model, TSDPSO-SVM has certain limitations in terms of interpreting corresponding recognition results. In practical applications, being able to explain the model's results may be important for clinicians and patients. Therefore, further research and exploration are necessary to improve the model's interpretability.

In summary, the combination of the TSDPSO-SVM model and the multidimensional feature fusion method proposed in this study has certain limitations in the identification of FRI. Nonetheless, it still holds significant value in improving the accuracy and efficiency of the identification of FRI. Future research can focus on these limitations to enhance the performance and reliability of models related to the identification of FRI.

## Data availability

The raw data supporting the conclusions of this article will be made available by Jianmin CHEN, without undue reservation.
